# Assessment of Agricultural Drought Risk in the Lancang-Mekong Region, South East Asia

**DOI:** 10.3390/ijerph17176153

**Published:** 2020-08-24

**Authors:** Lei Zhang, Wei Song, Wen Song

**Affiliations:** 1 National Disaster Reduction Center of China, Beijing 100124, China; zhanglei@ndrcc.org.cn; 2 Key Laboratory of Land Surface Pattern and Simulation, Institute of Geographic Sciences and Natural Resources Research, Chinese Academy of Sciences, Beijing 100101, China; zcyswszq@gmail.com; 3 College of Mining Engineering, North China University of Science and Technology, Tangshan 063210, China

**Keywords:** natural disaster, agricultural drought risk, hazard, vulnerability, Lancang-Mekong Region

## Abstract

Natural disasters worldwide regularly impact on human activities. As a frequently occurring natural disaster, drought has adverse impacts on agricultural production. The Lancang-Mekong River is a transnational river running through China and five Southeast Asian countries and it is a vital water resource for irrigation in the region. Drought in the Lancang-Mekong Region (LMR) has occurred frequently in recent years. Assessing the risk of drought in the region is essential for rational planning of agricultural production and formulation of drought relief measures. In this study, an assessment of drought risk has been achieved by combining the hazard and vulnerability assessments for drought. The assessment of the drought hazard depends mainly on the standardized precipitation index (SPI). The assessment of drought vulnerability takes into account various indicators such as climatic factors (e.g., crop water stress index), soil factors (e.g., available water capacity), and irrigation factors (e.g., irrigation support). The results reveal that: (1) Drought distribution in the LMR is characterized by a spreading of the drought to countries along the middle and lower reaches of the Mekong River. Countries located in the middle and lower reaches of the Mekong River are more prone to drought. Laos, Thailand, and Cambodia are the regions with higher and high-drought risk levels. (2) The spatial distributions for the drought hazard and the drought vulnerability in the LMR exhibit significant differences as evidenced in the mapping results. High-hazard and high-vulnerability areas are mainly distributed in the middle LMR, and the middle to higher hazard areas and the middle to higher vulnerability areas are mainly distributed in the south-central LMR, while the low-hazard areas and the low-vulnerability areas are mainly in the north. (3) The majority of planting areas for sugarcane, rice, and cassava are located in the high-hazard areas. The distributions of drought-prone and high-hazard areas also correspond to the main agricultural areas in the LMR.

## 1. Introduction

For many years, natural disasters throughout the world have regularly caused serious impacts to life and societies [1,2]. Natural disasters often result in the loss of natural and social resources [3,4]. According to the types, intensity, and space–time distribution of disasters, the risks and losses caused by natural disasters may be different [5,6,7]. Although the occurrence of natural disasters is inevitable, they can be reasonably anticipated by studying their nature and historical data in order to provide for possible early warning, effective monitoring, and prevention [4,8]. Therefore, scientific assessment of natural disaster risk, together with subjective judgement, can effectively strengthen the risk management and mitigation measures for disaster prevention.

Drought is one of the most important types of natural disasters [9]. It is a complex and multi-faceted disaster caused by natural and human factors [10], and has the characteristics of high frequency, large influence, long duration, and great harm [11]. The Intergovernmental Panel on Climate Change (IPCC) has stated [12,13,14] that the future risk of agricultural drought is increasing globally. At the same time, with a more frequent occurrence of drought, its potential threat to agriculture in developing countries is also increasing [15,16,17,18]. Agriculture is one of the areas that is heavily affected by drought [19,20,21]. Agricultural drought will have a series of negative effects on food production, people’s lives, and the ecological environment [22,23]. For example, the decline of precipitation in the north-east of the United States in 2016, and the fact that summer temperatures exceeded historical peaks, resulted in a 30% reduction in the yield per unit of most agricultural and pastoral products in the region, with some losses even reaching 90% [24]. Affected by a combination of climate, social production, and political factors, drought in Uganda has become like a “war” cycle, making agriculture land untillable, and where people have chronic hunger and the ecological environment is severely damaged [25]. A scientific risk assessment can help to provide effective disaster prevention and mitigation guidance in the case of agricultural drought and reduce the losses caused by drought [26,27].

In the early 1980s, researchers began to pay attention to the factors responsible for the formation of natural disasters and risk assessment theory, and the interaction of natural disasters and risk elements and the role of mathematical expressions in the subject matter were discussed [28,29,30]. These efforts stimulated the development of natural disaster risk theory such that agricultural drought risk research has also been greatly developed [31,32,33]. At present, the formation mechanism for agricultural drought risk is dominated by “factor theory” [33]. For example, researchers have identified three aspects of the drought index in risk assessment, namely, the hazard, the exposure, and the vulnerability elements, considering indices such as agricultural drought, irrigation, and the social ecosystem. Using this framework, information on agricultural drought risk at a global scale was analyzed [23]. By considering a variety of factors, the research was able to predict the agricultural drought risk in Africa from the years 2010 to 2100 [34]. In addition, based on the hazard and vulnerability factors, researchers also constructed a comprehensive risk model for drought and evaluated the distribution of drought risk in southwest China [35]. Furthermore, stability factors were constructed based on the meteorological drought conditions for a 10-year scale, and the drought risk of different states in the United States from the year 1901 to 2017 [36] was analyzed. These factor-led drought risk analyses and assessments have been recognized by many scholars and have provided effective help for governments to defend against drought disasters [37]. Therefore, “factor-led” agricultural drought risk assessment is an effective way to reduce losses through drought.

The Lancang-Mekong Region (LMR) spans six countries and regions in Southeast Asia. The Lancang-Mekong River is a vital water resource for irrigation in the LMR. In 2016, China, Cambodia, Laos, Myanmar, Thailand, and Vietnam jointly launched and built a new sub-regional cooperation mechanism, which made the region cooperate more closely on food production security and other food-related issues. Today, the LMR continues to be affected by floods and drought [38,39]. Among the countries in the LMR region, the impacts of drought on agricultural production are particularly serious, so drought risk assessment in the region is essential [39,40,41]. Some researchers have used the Normalized Difference Vegetation Index (NDVI) and the Land Surface Temperature (LST) to calculate the Temperature Vegetation Dryness Index (TVDI) to assess agricultural drought in Laos, Thailand, Cambodia, and Vietnam [40]. The Standardized Precipitation Index (SPI) has also been used to analyze historical drought data in the lower Mekong basin from 1965 to 2006, and this enabled the drought risk for the years 2016 to 2099 to be estimated [42]. Furthermore, based on the natural disaster risk theory, the probability of drought occurrence in Yunnan province, China has also been evaluated [43]. The studies mentioned above [39,40,41,42,43] indicate that there has been some research on drought risk assessment in the LMR, but most of the studies have been limited to local regions, and the studies have lacked a holistic approach to drought risk assessment; moreover, the spatial distribution of risk is unknown.

A major aim of this paper is to redress the lack of a holistic assessment of drought risk across the LMR. Specifically, this study is concerned with the following: (1) Using the SPI to assess the hazard of agricultural drought in the LMR; (2) selection of evaluation indicators associated with the climate, soil, and irrigation to facilitate comprehensive analysis of the agricultural drought vulnerability in the LMR; (3) assessment of the agricultural drought risk in the LMR based on the hazard and vulnerability assessments. The paper is arranged as follows: In Section 2, an overview of the study area and descriptions and sources of the study data are given; in Section 3, the method for assessing agricultural drought risk is introduced; in Section 4, the results for agricultural drought risk are presented; and main findings are discussed in Section 5.

## 2. Study Area and Data Sources

### 2.1. Overview of the Study Area

**Figure 1:**
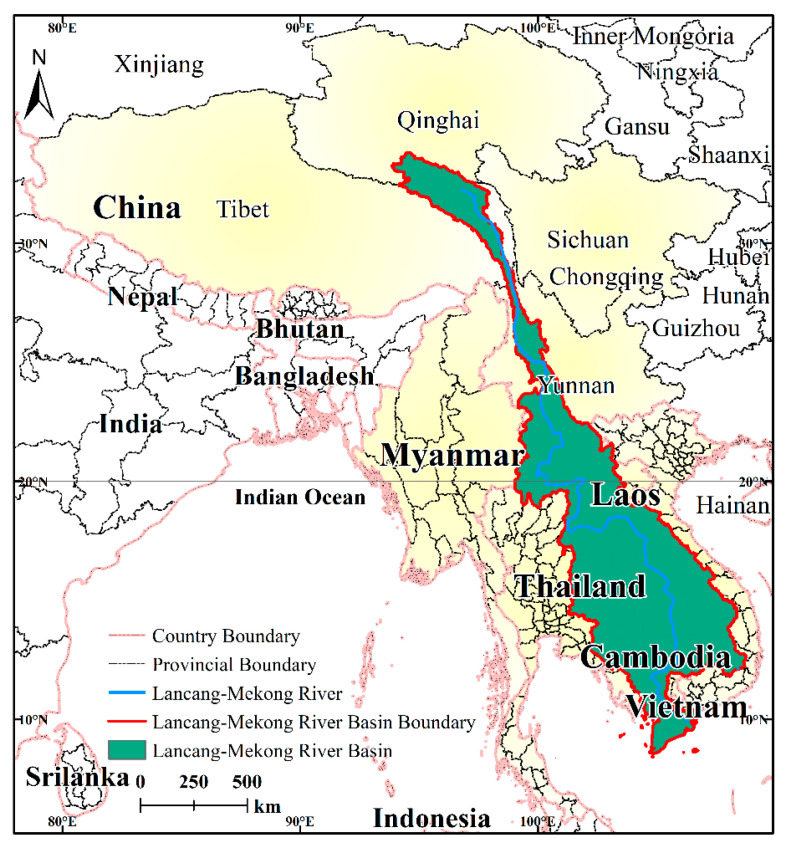
Geographical location of the Lancang-Mekong Region (LMR).

The Lancang-Mekong River, which flows through the four provinces of Qinghai, Tibet, Sichuan, and Yunnan in China, as well as Myanmar, Thailand, Laos, Cambodia, and Vietnam, is called the Lancang River in China and the Mekong River in the five countries of Southeast Asia (Figure 1). The LMR (the four provinces in China and the five countries in Southeast Asia) lies between 13°45′ N, 78°25′ E and 39°19′ N, 109°29′ E, has a total area of 5 million km^2^, and serves more than 400 million people. The natural landscape of the river basin is diverse, covering a variety of climatic types, e.g., cold temperate zone, temperate zone, subtropical zone, and tropical zone. There are many geomorphological types in the LMR, such as glacier, alpine mountain area, plateau meadow, deep mountain range, shallow hill, alluvial plain, and estuarine delta. The whole region, especially the sub-region (five countries of Southeast Asia and Yunnan Province in China), has good natural conditions including plentiful water, hydro-electric, biological, and mineral resources, with great economic and development potential.

### 2.2. Data Sources and Preprocessing

#### 2.2.1. Precipitation Data

The precipitation data used in the study were the daily precipitation data with a spatial resolution of 0.25° × 0.25° (° stands for degree) and were obtained from the global land surface data assimilation system (GLDAS) for the period 1965–2014 [44]. The mask environment of the LMR was utilized to obtain precipitation data using the ArcGIS extraction function.

**Figure 2:**
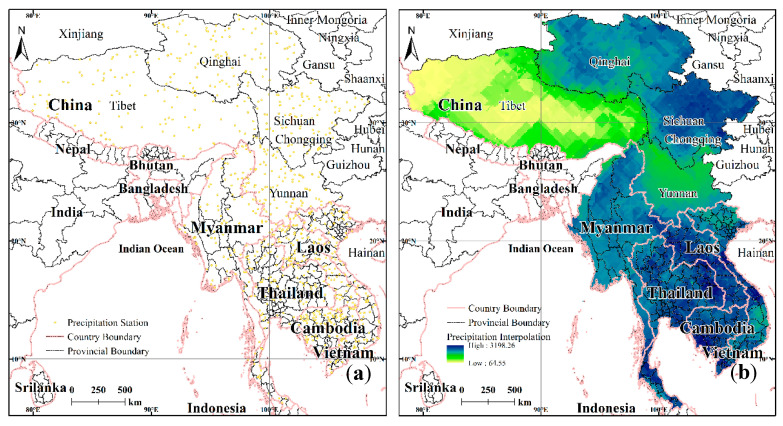
Distribution of sites (**a**) and precipitation (**b**) in the LMR.

Given the large amount of raw precipitation data and the fact that visualization itself was not conducive to large-scale research evaluation, the precipitation rate attribute values for the data were extracted as point data using a Python script, resulting in the LMR 900 base site data being obtained (Figure 2a). Processing of the site observation data proceeded as follows: (1) The data were checked to revise the part site location and elevation information. (2) The missing partial site data were processed. For data missing for 1–2 days, the interpolation process was performed using the value of the similar period before and after the days in question. For data that were missing for more than 10 consecutive days, the values of the site for that period were taken as invalid and were not involved in later processing operations. (3) MATLAB was used to collate data and facilitate interpolation. The location information for the site was accurate to the point (“), with the elevation information accurate to 0.1 m; the units for the temperature data were accurate to 0.1 °C, and the precipitation data were accurate to 0.1 mm.

On the basis of previous studies and comparative analysis of the interpolation results of the meteorological data using various interpolation models, it has been shown that the spatial correlation model ANUSPLIN gave the best fit for the spatial distribution of the meteorological elements [45]. The optimized thin-plate smooth spline function was used to fit the meteorological data, and there was no limit on the number of meteorological stations needed. Therefore, the ANUSPLIN model was selected for interpolation to obtain the distribution of the precipitation data [46]. The model requires the input of the location and elevation of the meteorological stations and other auxiliary data, as well as factor values. The common equation of the thin-plate spline function is given elsewhere [47].

#### 2.2.2. Evapotranspiration

**Figure 3:**
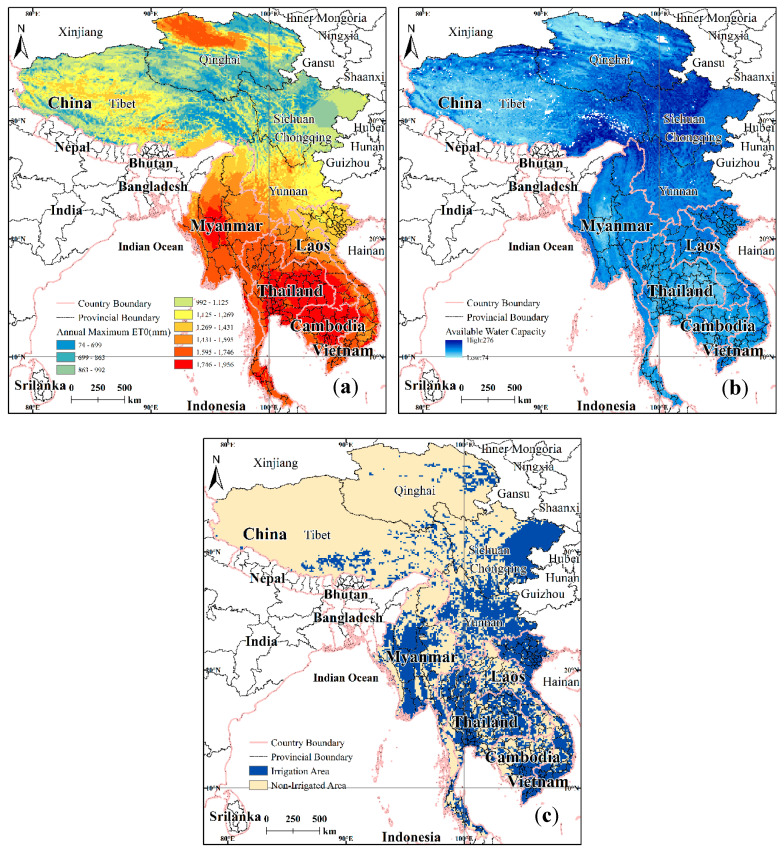
Evaporation of reference crops (**a**), available water capacity (**b**), and irrigation distribution (**c**) in the LMR.

The evapotranspiration data are derived from the Global-Aridity index (Global-Aridity_ET0) and the global reference evapotranspiration (Global-ET_0_) dataset. The Global-ET_0_ dataset provides high-resolution (30 arcsec) global raster climate data [48,49] for the period 1970–2000 associated with evapotranspiration and rainfall. The Penman–Monteith formula, as recommended by the Food and Agricultural Organization (FAO, 1998) of the United Nations, was applied to calculate the *ET*_0_ (reference crop water requirement) values [50]. The maximum possible annual mean for the evapotranspiration for the LMR was from 74 to 1956 mm, as shown in Figure 3a. Most of the areas shown range from 800 to 1300 mm and 1500 to 1800 mm, while evapotranspiration values for the Hengduan Mountain areas are mostly low; evapotranspiration values for the northwest of Qinghai, the central part of Myanmar, eastern Thailand, and northern Cambodia are above 1746 mm, which correspond to the high-evapotranspiration areas of the LMR.

#### 2.2.3. Available Water Capacity

The available water capacity (AWC) data are derived from the global soil hydraulic parameters—soil physics hydraulic background parameters and termed “Global Map”, which were studied jointly by Tianjin University and the University of Arizona [51]. The data are raster data with a resolution of 1 km × 1 km (Figure 3b). Finally, the data were resampled in ArcGIS at a resolution of 10 km × 10 km.

#### 2.2.4. Agricultural Irrigation and Zone Data

The global irrigation area map (Global Map of Irrigated Areas-version 5.0, GMIA-v5.0) is published by the FOA [52]. The data are in raster format and the resolution is 10 km × 10 km. The irrigation distribution data for the LMR were extracted based on the administrative divisions of the LMR (Figure 3c). The agricultural irrigation areas of the LMR are mainly concentrated in the plain terrains between mountainous regions, such as the central part of Myanmar, the northern part of Vietnam, and the eastern part of Sichuan, where there are intensive irrigation areas. The northern irrigation areas of the LMR are scattered, for example, the eastern part of Qinghai and the southeast of Tibet. From the statistics, the irrigation areas accounted for 34.9% of the LMR.

#### 2.2.5. Crop Phenology and Zone Data

The crop phenology data (growth stages and growing seasons for sugarcane, rice, and cassava) were derived from the World Food Program’s crop water-requirements guidance document (Table 1) with reference to the FAO-56 standard [53]. Sugarcane, rice, and cassava are of the three main crops in the LMR [54].

**Figure 4:**
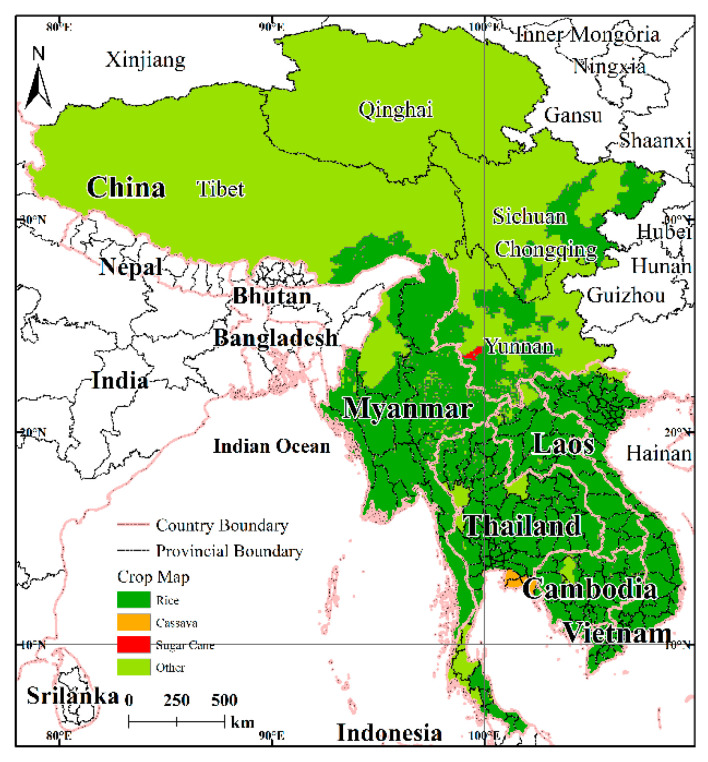
Distribution of crops in the LMR.

The data for the agricultural zones are from the global crop distribution dataset [55]. The spatial resolution of the data is 10 km × 10 km. The data describe the global crop census data (harvest area and yield) from 1997 to 2003. The spatial distributions for sugar cane, rice, and cassava in the LMR were obtained under the auspices of the administrative divisions of the LMR (Figure 4).

#### 2.2.6. Historical Drought Data

The historical drought information for the LMR covers five countries in South-East Asia and four provinces in China, and is based on the Global Disaster Database (EM-DAT), supplemented by the Desinventar Database, the Reliefweb, and the ADRC (Asian Disaster Reduction Centre). A total of 35 data sets were obtained for the period 1965–2017. These data sets relate to Thailand (9), Cambodia (6), Sichuan (6), Vietnam (5), Laos (4), Yunnan (4), Qinghai (1), Myanmar (0), and Tibet (0) (figures in brackets refer to the number of times data were accessed).

## 3. Research Methods

### 3.1. Framework for Agricultural Drought Risk Assessment

**Figure 5:**
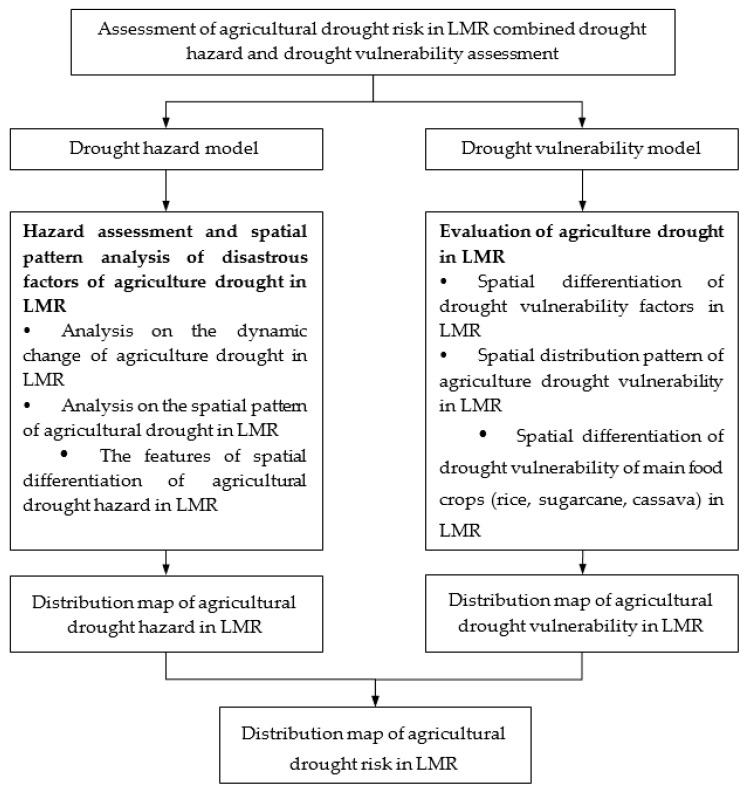
Agricultural drought risk assessment process for the Lancang-Mekong Region (LMR).

The main research steps for agricultural drought risk assessment in the LMR are outlined in Figure 5 and consist of the following: (1) Proposing the drought risk assessment method by combining the hazard and vulnerability assessments; (2) developing a database of risk assessment at a resolution of l0 km × l0 km; (3) analyzing the spatial distribution of the drought hazard and the vulnerability; (4) evaluating the risk of drought in the LMR and identifying the spatial distribution pattern to provide a scientific decision basis for drought risk management.

### 3.2. Assessment of Drought Hazard

**Figure 6:**
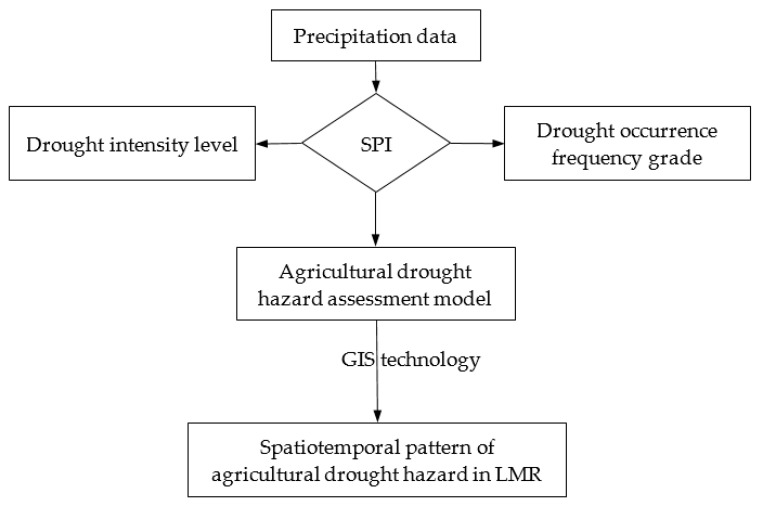
Drought hazard assessment methodology.

The intensity of drought depends on the degree of the water deficit, and the duration and the spatial range of the impact [56,57]. The use of SPI analysis for drought characterization and drought hazard assessment is widely recognized [58,59,60]. The main advantages of the SPI are mainly reflected in two aspects [61,62]: (1) The calculation of the SPI only needs rainfall data, and is not affected by topographic factors; thus, it is convenient to carry out comparative spatial research. (2) The SPI’s multi-time-scale features enable calculations to be performed at different time-scales, and the approach can be used not only to monitor water changes in a short period of time, but also to monitor water dynamics over a long time period. Given that water changes in a short period of time can have an important impact on agriculture yield, this study carries out hazard analysis and calculation of drought based on an SPI at a three-month scale. Based on historical rainfall data, the intensity characteristics of drought are identified by the SPI, and the frequency of drought occurrence in different regions is calculated statistically. Then, the drought hazard assessment model, which reflects comprehensively the drought intensity and frequency, is constructed, and the spatial distribution characteristics of the drought hazard are able to be discussed based on the results of the model calculation (Figure 6).

The SPI method is based on the use of the Γ probability distribution function to fit the historical rainfall time series for each station [58], that is (Equation (1)):

(1) $$g\left(x\right)=\frac{1}{\beta^{\alpha }\Gamma \left(\alpha \right)}\chi^{\alpha -1}e^{-x/\beta }$$

When x > 0, it represents rainfall; when α > 0, it represents the shape parameter; when β > 0, it represents the scale parameter; and Γ(α) is the gamma function.

The MLE (maximum likelihood estimation) method is used to obtain the value of α and β (Equations (2)–(4)):

(2) $$\alpha =\frac{1}{4A}\left(1+\sqrt{1+\frac{4A}{3}}\right)$$

(3) $$\beta =\frac{\bar{x}}{\alpha }$$

(4) $$A=\ln\left(\bar{x}\right)-\frac{\sum \ln\left(\bar{x}\right)}{n}$$

where n represents the length of the rainfall sequence. Thus, the cumulative probability of a given time-scale can be calculated as follows (Equation (5)):

(5) $$G\left(x\right)=\overset{x}{\underset{0}{\int }}g\left(x\right)dx=\frac{1}{\beta^{\alpha }\Gamma \left(\alpha \right)}\overset{x}{\underset{0}{\int }}x^{\alpha -1}e^{-x/\beta }dx$$

Given that the gamma function does not include the case that x is 0, and the actual precipitation can be 0, the cumulative probability is expressed as (Equation (6)):

(6) H(x)=q+(1−q)G(x)

where q represents the probability of zero precipitation. After that, the cumulative probability function H(x) is transformed into the standard normal distribution function with a mean value of 0 and a standard deviation of 1; then, the SPI is obtained.

H(*x*) is the cumulative probability (H(*x*) ∈ {0, 1}). For the standard normal distribution function, assuming F(SPI) = H(*x*), the value of SPI can be deduced by knowing H(*x*). For example, if the rainfall of a certain month *x* = 100 mm and the corresponding H(100) = 0.5, we can deduce that SPI = 0.

When SPI < 0, it represents drought and when SPI > 0, it indicates wetting. The commonly used SPI calculation scale parameter β is set to 1, 3, 6, 12, 24, and 36, which represent the SPI values with one month, three months, six months, one year, two years, and three years as the step size, respectively, and are counted as SPI(β). The World Meteorological Organization (WMO) has published standardized SPI guidance literature [63], and SPI(β) statistical results can be obtained as long as the corresponding format requirements are strictly adhered to. This study performs drought hazard analysis calculations at a three-month scale for the SPI (Table 2), using SPI (3) from the years 1975 to 2014.

According to the size of the SPI value, the drought condition was divided into 4 grades (Table 3).

The hazard of drought depends on the intensity and frequency of the drought. The greater the intensity and frequency of drought, the greater the hazard. Comprehensive drought hazard assessment models should be able to reflect comprehensively the multiple characteristics of drought. With reference to existing research results and the SPI results, drought of different intensity grades is given different weightings, and the incidence rate of each drought grade is calculated. The drought hazard model is constructed as follows (Equation (7)) [64]:

(7) $$\mathrm{DHI}=\left(MD_{r}\times MD_{w}\right)+\left(SD_{r}\times SD_{w}\right)+\left(VD_{r}\times VD_{w}\right)$$

where DHI represents the drought hazard index; $MD_{r}$ represents a moderate drought rate; $MD_{w}$ represents a moderate drought weight; $SD_{r}$ represents a severe drought rate; $SD_{w}$ represents a severe drought weight; $VD_{r}$ represents a very severe drought rate; $VD_{w}$ represents a very severe drought weight. The impact of mild drought on agricultural production is very small and, therefore, is not considered. The model takes into account the intensity and frequency characteristics of drought and the time-scale characteristics of the SPI, while the application of Geographic Information System (GIS) can describe the spatial distribution characteristics of drought.

Here, we used the scoring method to determine the weight according to the grade of probability of occurrence. When −1.5 < SPI < −1, the probability of the normal distribution function is 0.0918; when −2 < SPI < −1.5, the probability of the normal distribution function is about 0.0440; when SPI < −2, the probability of the normal distribution function is about 0.0227. The probability of occurrence of the three ranges is approximately proportional, with a ratio of 0.5. When scoring, every time the probability of occurrence is halved, the weight increases by one level, so the corresponding weights are 1, 2, and 3, respectively.

### 3.3. Assessment of Drought Vulnerability

#### 3.3.1. Selection and Method for Calculation of the Drought Vulnerability Factor

**Figure 7:**
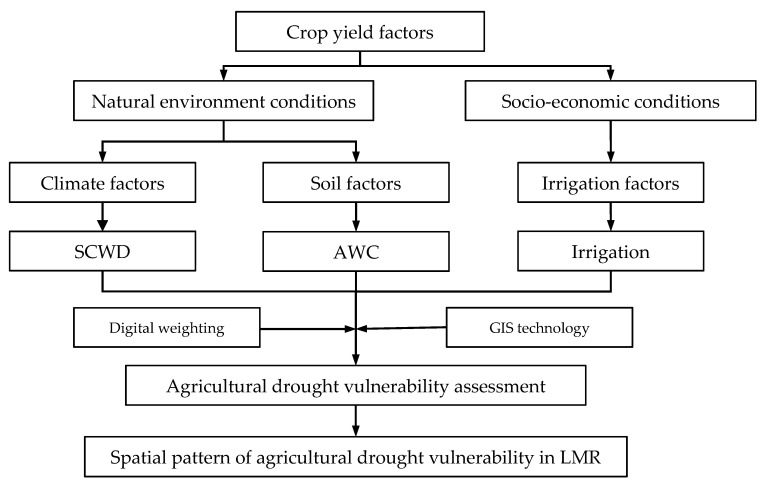
Agricultural drought vulnerability assessment process.

The vulnerability was assessed based on the vulnerability evaluation of natural environmental conditions and socio-economic conditions of crop production. Aspects of the three key factors (climate, soil, and irrigation) closely related to the production of major crops in the LMR were considered for vulnerability assessment index selection (Figure 7). For the factors of climate, soil, and irrigation, many indices can be selected for vulnerability assessment. Nevertheless, the data of some indices cannot be obtained in LMR. Referencing the previous research and the data availability, the water deficiency rate, the AWC, and the availability of irrigation in the crop growing season were selected as the specific indices for vulnerability assessment. Given that the vulnerability to drought in the LMR is affected by the above three factors, a conceptual model for drought vulnerability assessment was constructed as follows (Equation (8)):

(8) V=G(f(C),f(S),f(I))

where V represents the agricultural drought vulnerability; f(C) represents the climate factor function, f(S) represents the soil factor function, and f(I) represents the irrigation factor function.

##### (1) Climate factors

The water demand in the crop growing season and the precipitation for the corresponding growing season can be used to estimate the water deficiency during the whole crop growth period; thus, providing a scientific basis for improving the water use efficiency of farmland and developing efficient water-saving measures for agriculture. The formula is as follows (Equation (9)) [65]:

(9) $$SCWD=\frac{ET-P}{ET}$$

where *SCWD* is the seasonal crop water deficiency; *ET* is the crop growth season water demand; and *P* is the crop growth season precipitation. When *SCWD* is positive, this indicates that the crop growth season is short of water, whereas a negative value indicates that the precipitation in the growth season can meet the crop growth season water demand.

A unit of crop water demand is generally measured by the depth (mm) of the water layer consumed at a certain period or during full fertility. For this study, the crop growth season water demand was expressed by the crop coefficient-reference crop water demand method (Equation (10)) [50], namely:

(10) $$ET=ET_{0}\cdot Kc$$

where $ET_{0}$ is the reference crop water requirement and Kc is the crop coefficient.

At the regional scale, the average crop water demand in the growing season is calculated on the basis of the weight of the crop planting area, that is (Equation (11)):

(11) $$\bar{ET}=\frac{ET_{w}*\rho_{w}+ET_{c}*\rho_{c}+ET_{r}*\rho_{r}}{\rho_{w}+\rho_{c}+\rho_{r}}$$

where $\bar{ET}$ represents the regional average water consumption for the crop in the growth season; *ET_w_*, *ET_c_*, and *ET_r_* represent the water consumption for sugarcane, rice, and cassava, respectively, in the growing season; $\rho_{w}$, $\rho_{c}$, and $\rho_{r}$ represent the percentage of planting area for sugarcane, rice, and cassava planting, respectively, in the whole agricultural region.

The seasonal crop water deficiency (*SCWD*) is as follows (Equation (12)):

(12) $$\bar{SCWD}=\frac{\bar{ET}-P}{\bar{ET}}$$

As there are large differences in the agricultural planting structure and the planting systems in various regions for the whole LMR, it is difficult to consider the water deficiency of each crop in the growing season. Therefore, this study makes use of the water deficiency of three major food crops, namely, sugarcane, rice, and cassava in the growing season, as the climate vulnerability factors of agricultural drought.

##### (2) Soil factors

The AWC refers to the amount of water that the soil can store at a certain depth and can be used by plants. It is an inherent characteristic of the soil, which shows the water supply capacity of well-drained soil to vegetation [66]. It is generally considered to be a measure of the soil moisture between the field water-holding capacity (FC) and the permanent wilting percentage (PWP), that is, the difference between the field water holding capacity and the water content for a permanent wilting scenario.

##### (3) Irrigation factors

Irrigation is the main agricultural production measure to prevent and reduce drought, which is of great significance to increasing crop yield, guarantee water supply security, food security, increasing the farmers’ income, and improving the ecological environment. The study hypothesized that irrigation can effectively mitigate the impact of water deficiency on agricultural production when drought occurs, and that irrigated agricultural areas without irrigation have higher vulnerability compared to irrigated agricultural areas where irrigation is available.

#### 3.3.2. Drought Vulnerability Assessment Model

Referring to existing research [43,67], vulnerability factor weightings were set in the drought vulnerability assessments as shown in Table 4, and each vulnerability factor was ranked, weighting each level with values between 1 to 5, where 1 indicates the least impact on drought vulnerability and 5 indicates a significant impact on vulnerability. Meanwhile, with respect to the weightings, to reflect the differences between the factors, different weighting ranges were set according to the importance of each factor in agricultural production. For example, for agricultural production, the available water-holding capacity and the climate conditions are more important; hence, the climate factor weighting range was set to 2–5, and the soil factor weighting range was set to 1–4. To demonstrate the importance of irrigation in agricultural production, the weightings for irrigated and non-irrigated areas were set to 1 and 4, respectively.

Finally, the drought vulnerability assessment model was constructed as follows (Equation (13)):

(13) $$V=W_{awc}+W_{wd}+W_{ir}$$

where V refers to the agricultural drought vulnerability; $W_{awc}$ refers to the available water-holding capacity weight; $W_{wd}$ refers to the water deficiency rate weighting in the crop growing season; $W_{ir}$ refers to the irrigation factor weighting.

By using the ArcGIS space calculation function, the three vulnerability factor layers were superimposed, the weights were summed, and the vulnerability of each grid cell was calculated. Then, the numerical values were normalized, and the vulnerability was categorized from high to low by the natural breakpoint method, to realize higher vulnerability, high vulnerability, medium vulnerability, and low vulnerability areas; finally, the outcomes were mapped.

### 3.4. Assessment of Drought Risk

Regional drought risk is the result of a combination of the regional drought hazard, the regional natural environment, and socio-economic vulnerability [23,68,69]. Therefore, drought risk assessment includes drought hazard assessment and vulnerability assessment. Based on this, a conceptual model of agricultural drought risk assessment was constructed as follows (Equation (14)):

(14) R=G(f(h),f(v))

where R refers to the agricultural drought risk, f(h) refers to the agricultural drought hazard function, and f(v) refers to the agricultural drought vulnerability function.

In the latest research, the conceptual model of risk multiplied by vulnerability is usually used to calculate the risk, namely, risk = hazard × vulnerability (Equation (15)) [69]. Based on this relationship, the present study established the drought risk assessment model as follows:

(15) DRI=DHI×DVI

where DRI refers to the agricultural drought risk index, DHI refers to the drought hazard index, and DVI refers to the agricultural drought vulnerability index.

## 4. Results and Discussion

### 4.1. Comprehensive Assessment of Agricultural Drought Hazard

#### 4.1.1. Spatial Pattern of Drought Occurrence

**Figure 8:**
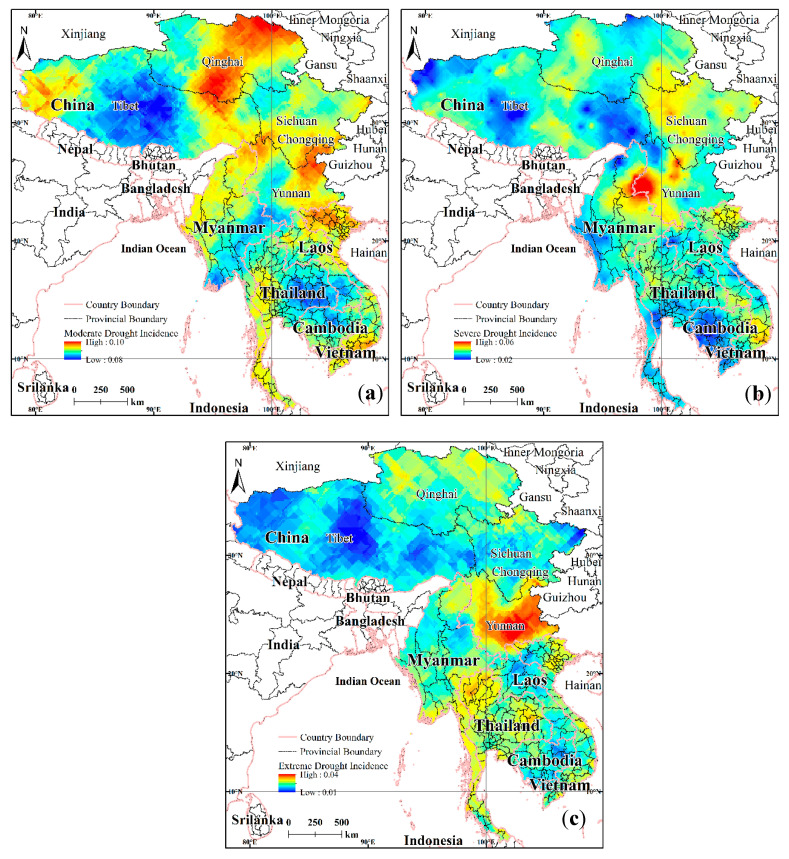
Spatial distributions for incidences of drought: (**a**) Moderate drought regions; (**b**) severe drought regions; (**c**) extreme drought regions.

The frequency of drought occurrence of varying intensities was analyzed using a three-month SPI scale with a 10 km × 10 km grid. The spatial distributions of the occurrence frequencies for (a) moderate drought, (b) severe drought, and (c) extreme drought within the LMR are presented in Figure 8. High incidence of extreme drought is mainly distributed in Yunnan Province, China and northwest Thailand. The central part of Yunnan Province is at the center of the distribution, and the extreme drought situation covers almost the whole of Yunnan Province. The high incidence of severe drought is mainly in the western Yunnan region, the western Sichuan region, Myanmar, northeast Laos, and the northernmost part of Vietnam. The high incidence of moderate drought is in northeast Yunnan, northern and southern Qinghai, southern Sichuan, western Tibet, Myanmar and western Thailand, coastal areas in southwest Cambodia, and coastal areas in central and northern Vietnam. Of these, only the moderate-drought-incidence regions of Vietnam and Cambodia are coastal with the rest being landlocked. In addition, Cambodia was least affected by the overall incidence of regional drought. Yunnan Province is the most LMR drought-prone region for all grades, and the province is also the drought center with respect to agriculture.

#### 4.1.2. Spatial Patterns of the Drought Hazard

**Figure 9:**
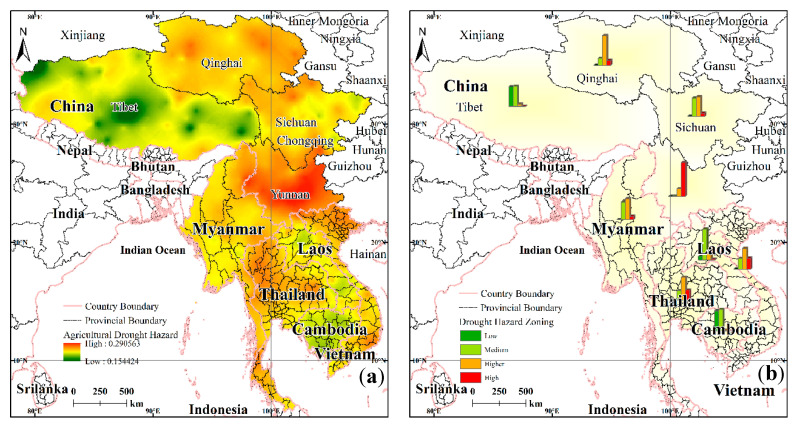
Spatial distribution (**a**) and regional differentiation (**b**) of the drought hazard in the LMR.

The drought hazard was calculated grid-by-grid, and was divided into four grades (low, medium, high, higher) from high to low by the natural breakpoint method. Thus, in this way, the distribution map of the drought hazard in the LMR was realized (Figure 9a). It can be seen that the high-hazard area is an area that has a high frequency and a high intensity of drought; the incidence of drought in the low-hazard area is low, and the drought intensity is also low.

The low-hazard areas are mainly distributed in the middle of Tibet, and account for 5.63% of the LMR area; in addition, most of the low-hazard areas are in plateau areas with very few crops. The medium hazardous areas account for 26.0% of the LMR area, and these areas are mostly in Cambodia and Tibet. The high-hazard zone, which accounts for 48.0% of the LMR area, is the largest area for all grades and is widely distributed throughout large areas of the LMR, such as Myanmar, Qinghai, and Sichuan, and these high-hazard zones are interspersed with the other hazard grades in Vietnam and Laos. The high-hazard zone means that the frequency or intensity of agricultural drought in the specific area is high, and will have a great influence on agricultural production. The higher-hazard zone is a region that experiences a higher frequency and intensity of drought (relative to a high-hazard zone), accounting for 20.4% of the LMR area. As can be seen in the distribution map, several high-value drought hazard centers in the LMR are located in the vast areas of the Yungui Plateau in China (e.g., Yunnan) and there is also a large distribution in northern Vietnam, western Thailand, and eastern Qinghai.

The distribution maps for sugarcane, cassava, and rice were extracted from the global crop distribution data and the drought hazard maps of the LMR. Statistical analysis shows (Table 5) that for the three food crop distribution areas, 38% of the sugar cane growing areas are located in high-hazard areas, while the corresponding values are 30% for cassava and 24% for rice. These three main crops are located mostly in the high-hazard areas. As can be seen from the above analysis, the drought hazard for agriculture in the LMR corresponds to the higher category.

#### 4.1.3. Comparative Analysis of the Drought Hazard in the Various Agricultural Areas

There are great differences in the drought hazard throughout the various agricultural areas of the LMR. Figure 9b is a bar chart of the proportion of drought hazards of different grades in each sub-region. Qinghai Province, China, is dominated by higher hazards, as is Yunnan Province; the Tibet Autonomous Region is dominated by lower and medium hazards, Sichuan Province by medium and higher hazards, and Laos by medium hazards. Myanmar is dominated by medium and higher-hazards as is Sichuan Province. Thailand mostly experiences higher-hazards, with some medium and high-hazards like Vietnam. Cambodia is similar to the Tibet Autonomous Region with respect to drought hazard. Overall, for the whole LMR, there are clear differences in the regional drought hazard but there are no obvious north–south or east–west trends or features.

### 4.2. Comprehensive Assessment of Agricultural Drought Vulnerability

#### 4.2.1. Drought Vulnerability Factors for the Various Regions

**Figure 10:**
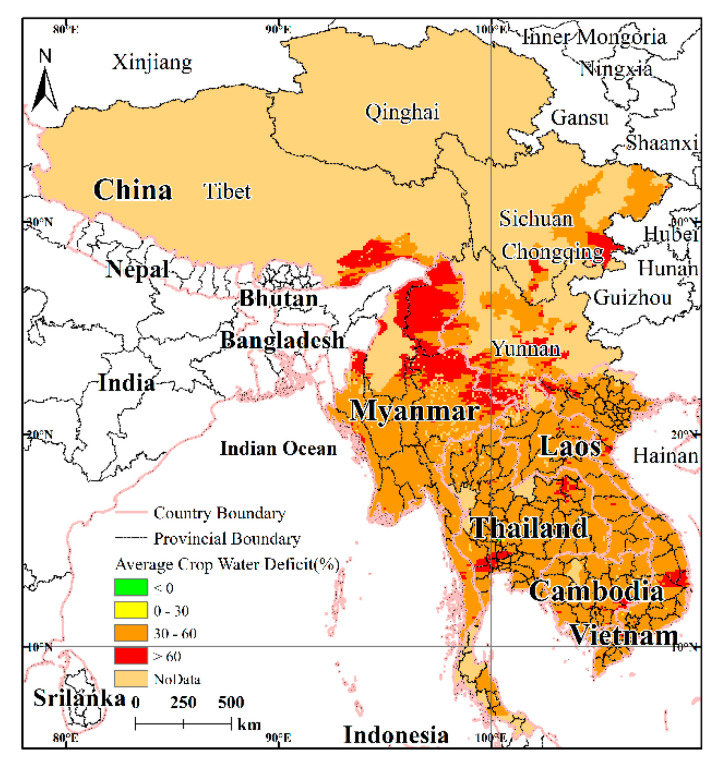
Water deficiency in the average growing season for crops.

Based on the main crop phenology and the crop coefficients, the average water demand values for rice, sugarcane, and cassava during the growing season were calculated. The average water demand for rice was 4609.10 mm, while the proportion of the planting area was 40.6%. The average water demand for sugarcane was 3012.20 mm, while the proportion of the planting area was 0.26%. The average water demand in the growing season for cassava was 3470.99 mm, while the proportion of the planting area was 0.07%. The results for the vulnerability factor calculations show (Figure 10) that the average water deficiency rates for the three crops in the growing season vary greatly from north to south and from east to west. In general, large areas in the south correspond to low-value areas of crop water deficiency, and the more one goes northward, the more the crop water deficiency rate increases.

#### 4.2.2. Analysis of Spatial Patterns of Drought Vulnerability

**Figure 11:**
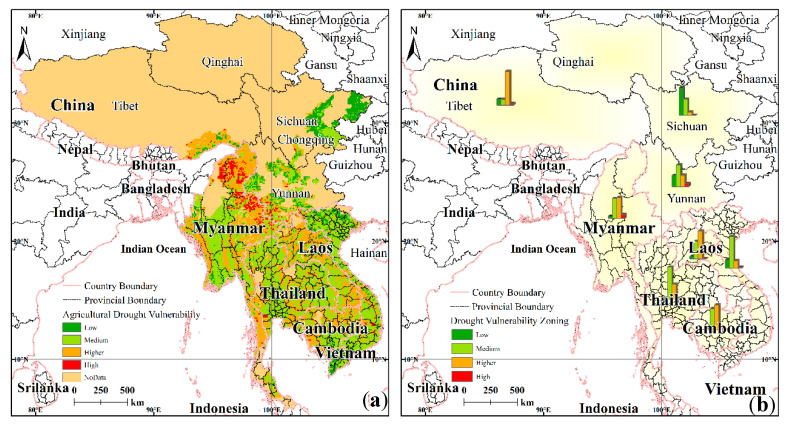
Spatial distribution (**a**) and regional differences (**b**) of drought vulnerability in the LMR.

The spatial distribution of drought vulnerability with respect to agriculture in the LMR exhibits clear spatial differences, as shown in Figure 11a. The low-vulnerability areas are mainly distributed in the northeast of the LMR, while the middle- and higher-vulnerability areas are mainly distributed in the southern part of the LMR and are intermixed with other regions of different vulnerability grades. The high-vulnerability areas are mainly distributed in the central part of the LMR.

The low-vulnerability zone is mainly distributed in the eastern part of Sichuan and the northern part of Vietnam, and the overall distribution range is small; this zone is the area with the least grain planting. The medium-vulnerability zone is the largest area of food cultivation, and this zone is affected by the terrain, which consists of the main mountainous flat areas, such as the central part of Myanmar, western Thailand, southern Cambodia, and other areas. The higher-vulnerability areas are mainly distributed in the northern, eastern, and western coastal areas of Myanmar, and the northern regions of Laos, which are mostly plateau and mountainous areas. High-vulnerability areas are located mainly in northern and northeast Myanmar, as well as southwest Yunnan.

#### 4.2.3. Comparative Analysis of Drought Vulnerability for the Various Regions

The fraction of area under drought vulnerability conditions in the various regions of the LMR is illustrated in Figure 11b. Combined with Table 6, it can be seen that there are significant differences in drought vulnerability between the different agricultural areas. At the same time, within each area, the spatial distribution of the drought vulnerability levels for the various agricultural areas is also quite different. From a regional perspective, the Tibet Autonomous Region is dominated by a higher-drought-vulnerability area, while Sichuan Province is dominated by a low-drought-vulnerability area, and Yunnan Province is more balanced having mostly a medium-drought vulnerability. Myanmar is dominated by moderate- and high-vulnerability areas. Thailand is dominated by moderate-vulnerability areas and with a few higher grades. Laos has mainly high-vulnerability areas and some medium-vulnerability areas. Cambodia is dominated by higher- and moderate-vulnerability areas. Vietnam is mostly dominated by moderate-vulnerability areas.

### 4.3. Drought Risk Assessment and Spatial Analysis Patterns

#### 4.3.1. Results for Drought Risk Assessment

**Figure 12:**
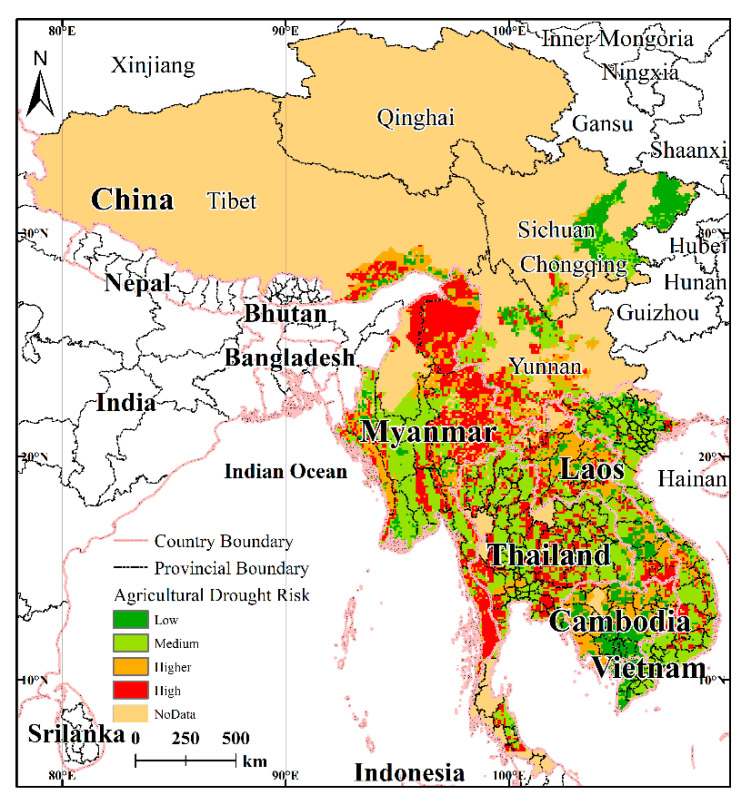
Distribution of drought risk in the LMR.

Spatial superposition calculations were performed using the results for drought hazard assessment and the vulnerability assessment. The risk assessment model was then constructed. Next, the GIS spatial analysis function was used to calculate the results for the drought risk assessment, and the natural breaks were used to classify the drought risk into four grades from the lowest to the highest. In this way, the spatial distribution pattern of drought risk in the LMR was revealed (Figure 12).

#### 4.3.2. Spatial Analysis Patterns for Assessment of Drought Risk

The drought risk distribution for the LMR with respect to agriculture exhibited clear regional differences, the map for the whole of the LMR indicating a distribution pattern of high-risk areas in the central region, low-risk areas in the regions adjoining the central region, high-risk in southern areas, and low-risk in northern areas. The high-risk areas are located in the middle and the northern part of each agricultural division, and the low-risk areas are located in the adjoining areas and the southern part of each agricultural division. The low-risk zone reflects a combination of low-vulnerability and low-hazard, with several distinct low-risk zones apparent in Figure 12, namely southern Myanmar, southern Laos, eastern and southeast Sichuan, and southern Cambodia. In addition, there are sporadic low-risk distributions in Yunnan, China, central Laos, and northern and southern Vietnam.

As can be seen from the vulnerability and hazard distributions for each country, areas with high drought hazard, such as Yunnan, Vietnam, and Thailand, are covered correspondingly with low-drought-vulnerability areas, whereas Cambodia with a low-drought hazard has a higher drought vulnerability. In addition, the same characteristics are also evident with respect to the spatial distributions for drought hazard and drought vulnerability, such as Myanmar, which has a higher drought hazard and moderate-higher drought vulnerability. This is also the case with Laos and Sichuan Province.

#### 4.3.3. Comparison of Drought Risk for the Various Regions

**Figure 13:**
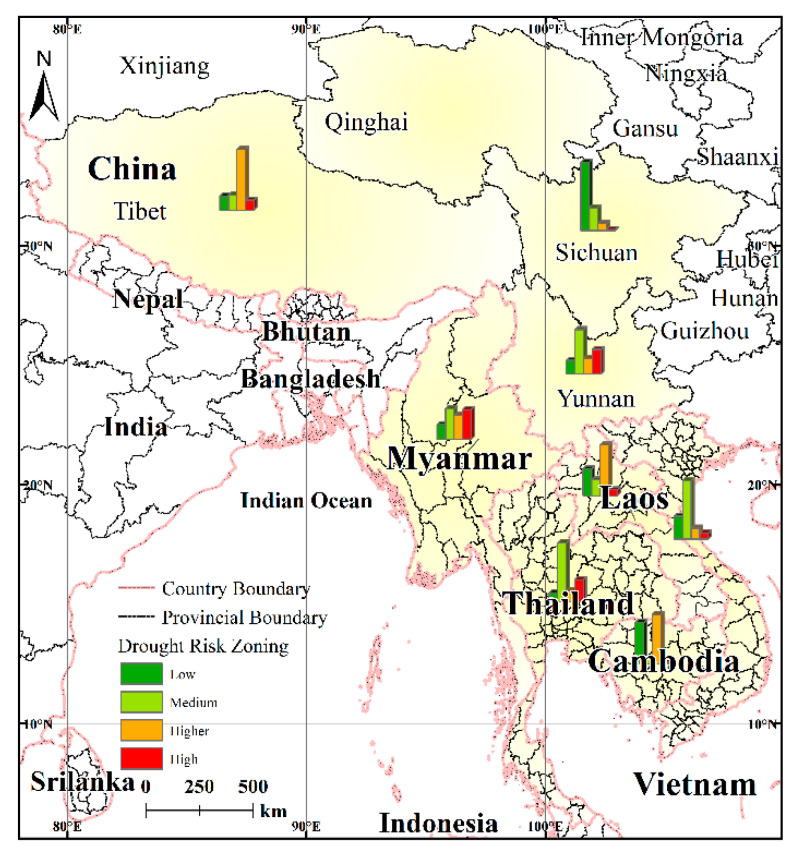
Regional differences in the agricultural drought risk in the LMR.

Compared to the zoning map for the LMR, statistical analysis of the drought risk in the various areas shows (Figure 13) that with respect to the drought risk, there are great differences between and within the various agricultural areas. Myanmar and Thailand, as well as Yunnan Province, have a relatively high proportion of high-risk regions, while Laos and Cambodia have a relatively higher proportion of high-risk levels, whereas Sichuan Province is almost all covered with low-risk areas. Compared to other countries, the drought risk in Yunnan is more balanced in terms of the spatial distribution with the different risk levels being relatively evenly distributed throughout the province.

In addition, the distribution of drought risk varies considerably within each agricultural area (Table 7). For example, the southern part of Myanmar is mainly low and medium risk as evidenced by a strip-like distribution and with some flake distribution, while the large area of drought risk in the north and northeast is flake distribution. The risk grade distribution in Thailand and Vietnam is similar, with both exhibiting a balance between high-risk and medium-risk distributions. Laos and the Tibet Autonomous Region are mainly low-risk and high-risk, respectively.

#### 4.3.4. Validation of Drought Risk Assessment

Drought disaster is a direct consequence of drought, so data for past drought disasters were used to validate the drought risk assessments for the LMR. Accordingly, based on the data for drought disaster in the LMR from 1965 to 2017, Thailand, Cambodia, and Vietnam were the main areas of drought disaster, and the drought risk levels in these areas were ranked as higher risk and high-risk. By comparison, the results of the present study for the regions that have suffered disasters in the past are also distributed mainly in the higher- and high-risk areas.

## 5. Conclusions

Based on assessment of the drought hazard and the drought vulnerability in the LMR, a conceptual model of agricultural drought risk assessment has been established. The higher and high-drought hazard areas are mainly distributed in large parts of Myanmar, Qinghai, Sichuan, and Yunnan, and in western Thailand. Among them, the plains areas, which have intensive agricultural production, are mainly located in the higher and high-hazard areas, and drought poses a great threat to agricultural production. Furthermore, there is a certain mismatch in the spatial patterns between the distributions of irrigation and drought hazard, which increases the risk of drought to some extent. The higher- and high-vulnerability drought areas are mainly distributed in the south-central LMR, particularly in the north-west, the north-east, northern Laos, and southwest Yunnan. The common feature of these areas is that during the growing season, the water deficiency rate is relatively high with a low irrigation rate.

The drought risk for the LMR has a distribution pattern of high-risk areas in the central region, with the adjoining areas being of low-risk, while the southern areas are high-risk and the northern areas are low-risk. Overall, the proportion of high-risk areas is 12.8% of the total, and these areas are distributed in the middle and northern part of each agricultural division. The higher-risk areas, the middle-risk areas, and the low-risk areas account for 28.8%, 31.7%, and 26.3%, respectively, of the drought-affected land. The results for the high-risk and the higher-risk drought areas were found to be consistent with the historical drought data for the LMR.

We propose four suggestions related to agricultural production activities and relief measures to minimize the drought risk. First, we recommend effective use and improve the related water conservancy projects, such as hydropower dams. These water conservancy projects can store water and divert floods, i.e., irrigating agriculture during dry periods and reducing the losses caused by floods. Second, it is suggested to adjust the crop plantation. Priority should be given to planting drought-tolerant crops in areas with high drought risk, and crops that require a lot of water to be planted in low-and medium-risk drought areas. Third, we recommend speeding up the selection of drought-tolerant crops. By planting crops with drought tolerance genes, water demand can be reduced. Lastly, we suggest applying modern technology to agricultural drought prevention and control, such as seawater irrigation and seawater crop planting (sea rice, etc.).

## Figures and Tables

**Table 1:** Growth season crop coefficients.

Crop Type	Early Growing Season	Mid-Growth Season	End of Growing Season
Sugarcane	0.40	1.25	0.75
Rice	1.05	1.20	0.75
Cassava	0.30	0.95	0.4

**Table 2:** Standardized precipitation index (SPI) (3) measurement.

Station	1975-01	1975-02	1975-03	1975-04	1975-05	2014-12
56100	−1.43	−0.57	−0.84	1.89	1.6	−0.31
56101	0.09	0.56	0.83	−0.16	−0.01	0.49
56102	0.68	0.36	0.09	−0.55	0.45	−0.56
56103	0.09	0.46	0.14	−1.11	0.7	−0.28
56104	−0.01	0.43	0.19	−0.7	0.65	0.45
56105	0	0.35	0.2	−0.93	−1.45	0.88
56999	−0.25	−0.43	−0.87	1.19	1.27	−1.21

Notes: The date in the table header is continuous data from 1975-01 to 2014-12; the station column in the table is continuous from 56100 to 56999. Due to limited space, only part of the data is displayed in the table.

**Table 3:** SPI (3) and classification of drought grades.

SPI (3) Value	Drought Level	Weightings	Incidence Rate
−1.0–0	Mild drought	-	-
−1.0–−1.49	Moderate drought	1	low
			medium
			higher
			high
−1.5–−1.99	Severe drought	2	low
			medium
			higher
			high
≤−2	Extreme drought	3	low
			medium
			higher
			high

**Table 4:** Weightings for the drought vulnerability assessment factors in the LMR.

Vulnerability Factor	Vulnerability Rating	Weightings
AWC	<100 mm	4
	100–175 mm	3
	175–250 mm	2
	>250 mm	1
$\bar{SCWD}$	<0	2
	0–30%	3
	30–60%	4
	>60%	5
Irrigation	Irrigated land	1
	Non-irrigated land	4

**Table 5:** Distribution of major crops for the different drought hazard grades in the LMR.

Drought Hazard	Main Crops		
	Sugarcane	Cassava	Rice
Low	0.00	0.00	0.00
Medium	0.06	0.08	0.21
Higher	0.56	0.62	0.55
High	0.38	0.30	0.24

**Table 6:** Regional differences of drought vulnerability in the LMR.

Area	The Fraction of Area under Drought Vulnerability Conditions				Regional $\bar{SCWD}$ During the Growing Season (%)	Regional Average AWC (mm)	Regional Average Irrigation Area Fraction
	Low	Medium	Higher	High			
Qinghai	0.00	0.00	0.00	0.00	0.0	166	0.07
Tibet	0.13	0.12	0.71	0.04	65.2	157	0.08
Sichuan	0.58	0.34	0.07	0.01	56.5	199	0.47
Yunnan	0.24	0.46	0.24	0.06	58.5	181	0.62
Myanmar	0.05	0.41	0.45	0.09	56.2	166	0.47
Thailand	0.01	0.67	0.31	0.01	52.8	147	0.66
Laos	0.05	0.36	0.58	0.01	54.5	165	0.39
Cambodia	0.02	0.44	0.52	0.02	51.8	149	0.43
Vietnam	0.18	0.66	0.15	0.01	53.3	166	0.85

**Table 7:** Regional differences of drought risk in the LMR.

Area	Proportion of Agricultural Risks By Region				High-Hazard Area Ratio	High-Vulnerability Area Ratio
	Low	Medium	Higher	High		
Yunnan	0.14	0.45	0.16	0.25	0.81	0.07
Cambodia	0.42	0.06	0.50	0.02	0.00	0.015
Laos	0.27	0.16	0.51	0.06	0.02	0.006
Myanmar	0.15	0.31	0.24	0.30	0.08	0.087
Thailand	0.09	0.59	0.11	0.22	0.24	0.008
Vietnam	0.23	0.59	0.11	0.06	0.26	0.014
Qinghai	0.00	0.00	0.00	0.00	0.12	0.000
Sichuan	0.69	0.23	0.07	0.01	0.08	0.003
Tibet	0.14	0.15	0.61	0.10	0.00	0.037
